# One-Step Approach to Treating Venous Insufficiency

**DOI:** 10.14740/jocmr2205w

**Published:** 2015-07-24

**Authors:** Farah Jarjous, Rafi Jarjous, George Nahhas

**Affiliations:** a Venocure, 2881 Monroe St, Dearborn, MI 48124, USA; b Section Cardiology, Oakwood Hospital and Medical Center, 18101 Oakwood, Dearborn, MI 48124, USA

**Keywords:** RFA, UGFS, DVT, Venous insufficiency, GSV, SSV, Reflux, Venous reflux disease

## Abstract

**Background:**

Patients with venous insufficiency can be treated with office-based, minimally invasive means like radiofrequency ablation (RFA) and ultrasound-guided foam sclerotherapy (UGFS). Traditional treatment involves ablation of the great saphenous vein (GSV) and the short saphenous vein (SSV) with RFA as a first step. The remaining refluxing tributaries are treated at a later session with UGFS or microphlebectomy. This approach is associated with an increased risk of thrombophlebitis while awaiting the second procedure. We, instead, elected to treat all the refluxing veins in one procedure which combines RFA of the truncal and perforating vein with UGFS to the accessory and tributary veins.

**Methods:**

A controlled non-randomized clinical trial, in which a total of 72 extremities were treated for vein incompetence in 63 consecutive patients aged 26 - 78 years, was conducted. Sixty-three extremities (87.5%) received treatment for reflux in GSV, 10 extremities (13.9%) were treated for reflux in SSV, and 11 (15.3%) were treated for reflux in the perforators. Reflux duration > 1 second to increase specificity and truncal vein diameter > 5 mm were identified in the treated limbs. The treatment was performed at our office and it involved delivering radiofrequency thermal energy to the truncal and perforating vein and then using foam sclerotherapy with the guidance of ultrasound to close the tributary and accessory veins in a single procedure. The results were monitored at 1 week and 6 weeks post-operatively by venous duplex ultrasound.

**Results:**

One hundred percent of the treated GSV and SSV and 91.7% of tributary veins were completely closed after the index procedure. Only 10 of 72 extremities (13.9%) needed a follow-up treatment to achieve closure of the perforator and accessory veins. By combining RFA with UGFS, our cohort did not experience thrombophlebitis or deep vein thrombosis (DVT) post-operatively. No major or minor complications were found upon follow-up evaluation.

**Conclusion:**

We believe that combining RFA with UGFS in a solo practice lowers the incidence of thrombophlebitis in the tributaries. Using this approach allowed us to achieve more complete resolution of venous reflux disease with lower complication rates in comparison with the popular staged strategy. This could have implications for financial savings to both the patient and the health system.

## Introduction

The most common treatment options for venous insufficiency are surgical stripping, microphlebectomy, endovenous thermal ablation with radiofrequency and laser therapy, and foam sclerotherapy. While surgical stripping reduces symptoms and improves quality of life, it is associated with postoperative bleeding, groin infection, thrombophlebitis, scarring, and nerve injury [1, 2]. It is costly and it requires an extended recovery time [1]. The other treatment options are less invasive and they relatively have the same success rates as the surgical approach, with the exception of ultrasound-guided foam sclerotherapy (UGFS) which has lower success rates as shown by Rasmussen et al [1]. Nevertheless, these latter treatment options are all office-based. They are lower in cost, have fewer complications, and allow for faster return to normal activities [3].

Currently, when confronted with a patient with venous insufficiency of the saphenous veins and their tributaries, the most common treatment strategy is to close the saphenous vein with endothermal ablation, and then weeks later, the remaining refluxing tributaries are treated with UGFS or microphlebectomy [4]. Although successful closure of great saphenous vein/short saphenous veins (GSV/SSV) by radiofrequency ablation (RFA) approaches 100% [5], this approach puts patients at risk for thrombophlebitis (3-20%) [6] in the untreated tributaries after closing the truncal vein. This, at least in theory, is due to blocking the outflow of blood from the tributary veins after closing the mother truncal vein. Needless to say, thrombophlebitis is painful, unsightly, and has the potential to propagate and cause deep vein thrombosis (DVT) and pulmonary embolus [7]. Moreover, the staged approach requires more resources, repeated duplex ultrasound, additional office visits and time off work; it also results in higher cost for the patient and insurers.

We hypothesized that treating all of the incompetent superficial veins in a limb in a one-step approach will have a comparable success rate and less complications, particularly thrombophlebitis and DVT, than the multi-step approach. We tested this idea by treating our cohort with RFA to the saphenous vein(s) and with UGFS to the tributaries in a single procedure. The success of the procedure was defined by the absence of reflux from venous duplex at 6 weeks post-procedure. Patient satisfaction was a secondary endpoint. This study was not randomized, but we used historical control for comparison. We did not identify any other study in the English literature that addresses this approach.

## Methods

### Demographics

We screened 72 consecutive patients (81 extremities) for eligibility based on the following criteria. Inclusion criteria: reflux duration > 1 s, and truncal veins with a diameter > 5 mm. Exclusion criteria: patients with history of phlebitis or DVT, and those who underwent venous ablation or sclerotherapy treatment.

Nine patients (nine limbs) were excluded for not meeting the inclusion criteria. The remaining 63 patients were treated for venous insufficiency in our office from April 2012 to November 2012. They all have symptomatic venous insufficiency with involvement of either or both saphenous veins and their tributaries, and/or the perforator veins. The 63 patients had vein incompetence in 72 limbs. The average age was 58 ± 13 years (range, 26 - 87 years) and 51 patients (70.8%) were women. The distribution of CEAP stages is shown in Table 1.

**Table 1:** CEAP Distribution of the 63 Patients (72 Limbs)

CEAP category	No. (%)
CEAP 1	3 (4.17)
CEAP 2	4 (5.56)
CEAP 3	60 (83.3)
CEAP 4	1 (1.39)
CEAP 5	2 (2.78)
CEAP 6	2 (2.78)

Of the 72 extremities, 61 had GSV reflux, eight had SSV reflux, two had insufficiency in both GSV and SSV, and one was treated for perforator reflux. There were also 10 extremities that had insufficiency in the perforators in addition to the truncal vein. Altogether, we treated 63 extremities (87.5%) for reflux in GSV, 10 extremities (13.9%) for reflux in SSV, and 11 (15.3%) for reflux in the perforators. Deep system reflux was found in three limbs (4.2%) and all 72 limbs (100%) had reflux in the tributaries. The presenting symptoms were as follows: pain in 70 patients (97.2%), edema in 60 patients (83.3%), and ulcer in four patients (5.6%).

### Treatment

All participating patients signed an informed consent allowing us to include them in this trial. The patients were treated in a solo practice by a single experienced operator using RFA to treat the incompetent truncal and perforating vein first, followed by UGFS injection in the tributaries of that vein. Meticulous attention was paid to close all the tributary and accessory veins during the injections. The patient was then rested for 10 min in Trendelenburg position.

The device used for RFA is ClosureFAST^TM^ (VNUS Medical Technologies, San Jose, CA, USA), and it was used as per manufacturer’s recommendation [8]. In brief, access of the vein was accomplished by puncture at a point that is distal to the reflux. A 7-F diameter catheter (ClosureFAST catheter) was inserted under the guidance of ultrasound and the tip was placed 2 cm from the saphenopopliteal or saphenofemoral junction to minimize the risk of DVT, while allowing for good efficacy. Tumescent anesthesia was applied, and then segmental heating to the saphenous vein with a 7-cm heating element was delivered. A heating temperature of 120 °C and cycle duration of 20 s was maintained. Each segment received two cycles of radiofrequency heat. The catheter was then removed and hemostasis was achieved. The tributary veins were subsequently treated with UGFS. The foam consisted of 0.75-1.5% solution of sotradecol (sodium tetradecyl sulfate): 1 mL solution was mixed with 4 mL air using a three-way stopcock as per Tessari’s method [9]. The treatment was performed while the patient was in supine position. We systematically injected the proximal and larger veins first with a 27-gauge needle, and then progressed distally. Ultrasound was used to guide foam injection and to prevent foam from reaching deep veins through perforators [10].

### Post-operatively

Graduated compression stockings 20 - 40 mm Hg thigh-high were applied before the patient was allowed to ambulate. Patients were instructed to wear the stockings continuously for 48 h, then during waking hours for 2 weeks. We prescribed ibuprofen (Motrin^®^) 400 - 800 mg three times daily along with frequent walking for 1 week. Venous duplex was done within a week of the procedure. At 6 weeks post-operatively, venous duplex ultrasound was repeated and a follow-up clinical evaluation was conducted to review the change in symptoms and the clinical appearance of the veins. The patients were instructed to call the office for any concerns or discomfort that may occur after the procedure. Additional face to face encounters were conducted when needed. The cohort was closely monitored for any evidence of thrombophlebitis or DVT. Thrombophlebitis was diagnosed by the presence of venous thrombosis, along with tenderness, erythema, and warm skin.

## Results

We were able to complete RFA followed by UGFS in all cohort. The average amount of foam used per procedure was 5.3 ± 2.8 mL (range 1 - 14 mL). At 6 weeks post-operatively, venous reflux was absent in all treated GSV/SSV and in all three patients (100%) who had deep system reflux. In the 11 patients with perforator vein incompetence, seven (63.6%) saw no reflux post-operatively. However, three patients, who had no perforator reflux pre-operatively, experienced it following treatment. With respect to the incompetent tributaries, only six of 72 extremities (8.3%) continued to have reflux post-operatively. Overall, 10 extremities needed a follow-up treatment to achieve closure of the perforator (seven patients) and accessory veins (eight patients).

**Figure 1:**
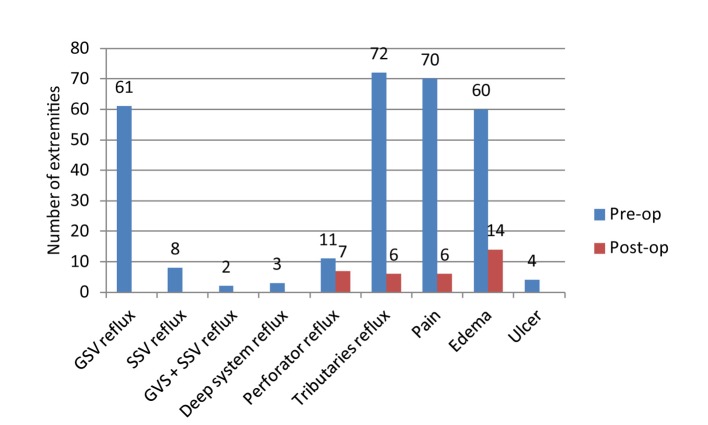
Assessment of reflux and symptoms pre-operatively and 6 weeks post-operatively.

Upon physical examination of patients 6 weeks after treatment, we found that edema was resolved in 46 of 60 patients (76.7%) and that ulcers healed in all three patients (100%) who had it pre-operatively. Of the 70 patients who experienced pain prior to the procedure, 64 (91.4%) reported, upon direct questioning, that their pain had vastly improved or completely resolved post-operatively. Figure 1 demonstrates a summary of these results.

None of the patients had thrombophlebitis, DVT, pulmonary embolism, or skin burns post-treatment. Our patients did not experience any minor complications such as hematoma, paresthesia, hemorrhage, or infections.

Telephone surveys were conducted to assess patient satisfaction, in which 61 of 72 patients participated. Of the 61 patients, 17 (27.9%) reported that their problems were completely resolved and 39 patients (63.9%) reported improved symptoms. There were four patients (6.6%) who reported no change post-operatively and one (1.6%) who experienced worsening in symptoms.

## Discussion

To date, we have not found a report in the English literature about combining RFA with UGFS in a single procedure to treat venous insufficiency. In this controlled clinical trial on 63 consecutive patients, we demonstrated that by treating all the refluxing veins in a limb in a one-step approach, we could achieve excellent success rate with negligible complications.

RFA is a minimally invasive technique that has high closure rates approaching 100% [5]. With RFA alone, subsequent treatment of the below-the-knee segment of the saphenous vein during some cases is often required [11]. Additionally, phlebectomy or UGFS is often performed upon follow-up visits to treat persistent refluxing tributary and accessory veins [8].

UGFS is most commonly used to close the tributaries. However, when UGFS is used as a solo treatment to venous insufficiency, Smith reported that closure rates of GSV and SSV are 88% and 83%, respectively [2]. Despite the success of UGFS, 48% of the treated extremities needed two treatments and 8% needed three treatments to achieve obliteration of the incompetent saphenous trunks and varices [2]. The reported average volume of foam sclerosant used per procedure is 8 mL (4 - 15 mL) [1].

By closing saphenous trunks, the outflow of the tributaries is blocked. This results in sluggish flow in them and increases the possibility of thrombophlebitis. After RFA, 3-20% [10] of patients experience thrombophlebitis and after UGFS, 14% [1] do. We treated the tributaries immediately after closing the truncal veins to eliminate the milieu for thrombophlebitis which explains our very low thrombotic complication rate.

To achieve closure of the tributaries, we used sotradecol 0.75-1.5% solution and the average volume per procedure was 5.3 ± 2.8 mL. This is lower than 8 mL solution of foam reported by Rasmussen et al who also reported using higher concentration of foam (3%) [1]. In our observation, the diameter of the tributary vein becomes significantly smaller right after closing the feeding truncal vein thus needing less foam for treatment. High volume of foam is directly related to complications like temporary visual disturbance, vasovagal fainting, as well as deep or superficial thrombosis [2, 12, 13]. Excessive browning of the skin may also be attributed to using more foam at a higher concentration. We strongly feel that by using the combined procedure, we were able to achieve as high of success rate as historically reported with less foam-related complications.

Other common complications that occur in the first few weeks include paresthesia, DVT, and hematoma. Paresthesia occurs in 4-20% [14] of patients treated with RFA and in 5-17% [1, 14] of those treated with UGFS. The rate of DVT is 0-16% [1, 11] in patients treated with RFA alone. On the other hand, only 1% of extremities undergoing UGFS treatment experience DVT [14]. Hematoma was observed in 12% of patients who underwent RFA to treat venous insufficiency [15]. When we used our one-step approach, we were able to achieve closure of 100% of the treated truncal veins and 91.7% of the tributaries while encountering none of these complications.

Although, the mechanism is not well understood, we believe that it could be related to shift in blood flow demands after closing the saphenous veins; three of our patients who had no prior perforator reflux pre-operatively, experienced it after treatment.

We did not conduct a financial analysis to study the impact of this combined strategy on cost; however, it is intuitive that it should have lower cost for the healthcare system, mainly due to less complications, procedures, duplex studies, and office visits which simplify patient care.

### Limitations

This study is observational and not randomized and it is subject to investigator and patient bias. We studied consecutive patients in attempt to avoid selection bias. The follow-up questionnaire was conducted by a third party oblivious to the nature of the procedure also to avoid operator’s bias. We did not have a control group, except for historical control, which makes this study more hypothesis-generating rather than conclusive. The follow-up was up to 6 weeks with very successful outcome. However, longer term outcome, studied through a larger randomized trial, is necessary for more firm establishment of this approach.

### Conclusion

In this single center experience, we demonstrated that combining RFA with UGFS in a single procedure is safe and successful in obliterating the entire refluxing vein. This approach has lower complication rate compared to the historical reports. A larger randomized trial comparing this approach with RFA followed by ancillary treatments is warranted.
